# Ca^2+^-sparks constitute elementary building blocks for global Ca^2+^-signals in myocytes of retinal arterioles

**DOI:** 10.1016/j.ceca.2006.08.005

**Published:** 2007-05

**Authors:** James Tumelty, Norman Scholfield, Michael Stewart, Tim Curtis, Graham McGeown

**Affiliations:** a Cell and Metabolic Signalling Group, School of Medicine and Dentistry, The Queen's University of Belfast, Medical Biology Centre, 97 Lisburn Road, Belfast BT9 7BL, Northern Ireland, United Kingdom; b Centre of Vision Sciences, The Queen's University of Belfast, Institute of Clinical Sciences, The Royal Victoria Hospital, Grosvenor Road, Belfast BT12 6BA, Northern Ireland, United Kingdom

**Keywords:** Ca^2+^-sparks, Ca^2+^-oscillations, Retinal arterioles, Ryanodine receptors, Smooth muscle, Sarcoplasmic reticulum, Vascular myocytes

## Abstract

Spontaneous Ca^2+^-events were imaged in myocytes within intact retinal arterioles (diameter <40 μm) freshly isolated from rat eyes. Ca^2+^-sparks were often observed to spread across the width of these small cells, and could summate to produce prolonged Ca^2+^-oscillations and contraction. Application of cyclopiazonic acid (20 μM) transiently increased spark frequency and oscillation amplitude, but inhibited both sparks and oscillations within 60 s. Both ryanodine (100 μM) and tetracaine (100 μM) reduced the frequency of sparks and oscillations, while tetracaine also reduced oscillation amplitude. None of these interventions affected spark amplitude. Nifedipine, which blocks store filling independently of any action on L-type Ca^2+^-channels in these cells, reduced the frequency and amplitude of both sparks and oscillations. Removal of external [Ca^2+^] (1 mM EGTA) also reduced the frequency of sparks and oscillations but these reductions were slower in onset than those in the presence of tetracaine or cyclopiazonic acid. Cyclopiazonic acid, nifedipine and low external [Ca^2+^] all reduced SR loading, as indicated by the amplitude of caffeine evoked Ca^2+^-transients. This study demonstrates for the first time that spontaneous Ca^2+^-events in small arterioles of the eye result from activation of ryanodine receptors in the SR and suggests that this activation is not tightly coupled to Ca^2+^-influx. The data also supports a model in which Ca^2+^-sparks act as building blocks for more prolonged, global Ca^2+^-signals.

## 1 Introduction

Although a considerable amount of research has been focussed on spontaneous Ca^2+^-events in smooth muscle, much of it has been carried out on isolated myocytes (see recent reviews [1–3]). It is increasingly recognised, however, that understanding the physiological integration of these events will require the study of intact tissues [4]. In systemic resistance arteries, for example, adrenergic stimulation can activate asynchronous Ca^2+^-oscillations within the myocytes of intact vessels, but no such responses are seen in cells isolated from similar vessels (see review [5]). Furthermore, due to the technical difficulties inherent in working with small blood vessels, relatively little information is available on the properties and mechanisms underlying spontaneous Ca^2+^-signals in terminal arterioles [4,6]. We have developed a retinal arteriole preparation in which the normal intercellular relationships within and between the endothelial and smooth muscle layers are maintained [7]. These small vessels represent the major resistance element within the retinal circulation and are vital to local control of blood flow [8]. The ever-increasing incidence of type 2 diabetes mellitus, with its retinal vascular complications, provides further motivation for this work (see reviews [9,10]), and it has recently been recognised that the structural changes of diabetic retinopathy are preceded by functional abnormalities, including loss of normal blood flow control [11]. This underlines the importance of understanding signalling within normal retinal arterioles.

Confocal Ca^2+^-imaging has previously revealed spontaneous Ca^2+^-sparks, oscillations and waves in smooth muscle cells embedded in the wall of intact segments of retinal arteriole [7]. Whilst these events have spatiotemporal characteristics similar to those in a range of other smooth muscles [1–4], the mechanisms responsible have not been investigated in this preparation. The functional significance of Ca^2+^-sparks also deserves further consideration. These may contribute to changes in cytoplasmic [Ca^2+^], and therefore contraction, either directly, or through feedback mechanisms dependent on activation of Ca^2+^-sensitive ion channels in the plasma membrane [3]. Although the potential for positive feedback through activation of Ca^2+^-sensitive Cl^−^ channels has been identified in vascular myocytes [12,13], sparks are usually ascribed an inhibitory role in systemic arterial smooth muscle [14]. By activating Ca^2+^-sensitive K^+^-channels, near-membrane sparks can cause hyperpolarisation, resulting in vascular relaxation secondary to the closure of voltage-activated Ca^2+^-channels. Evidence for such a negative feedback mechanism has now been found in a variety of tissues [15,16] and may, for example, represent an important signalling pathway during agonist dependent relaxation of urinary bladder [17,28]. Preliminary observations on retinal arterioles suggest, however, that Ca^2+^-sparks may play an excitatory role in these vessels, summating to produce prolonged, global Ca^2+^-oscillations and vascular contraction [7]. This important observation has been confirmed and extended in the current study, in which faster imaging was used to demonstrate that Ca^2+^-waves and oscillations often originate from spark sites. The mechanisms responsible for the spontaneous Ca^2+^-events seen have also been investigated for the first time. The results obtained, whilst confirming the well-recognised role of store release via ryanodine receptors (RyRs), also suggest that modulation of spark frequency can directly regulate both the amplitude and frequency of Ca^2+^-waves and oscillations, even when spark amplitude is unaffected.

## 2 Materials and methods

### 2.1 Retinal microvessel preparation

Animal use conformed to the requirements of The Animals (Scientific Procedures) Act (1986) and the principles set out in the Guide for the Care and Use of Laboratory Animals (National Academy of Sciences 1996). Male Sprague–Dawley rats (200–300 g) were anaesthetized with CO_2_ and killed by cervical dislocation. Retinae were rapidly removed and arterioles isolated as previously described [19,20]. In brief, retinal quadrants were lightly triturated using a fire-polished pipette in a low Ca^2+^ Hanks’ solution and centrifuged at 1200 × *g* for 1 min. The supernatant was aspirated off and the tissue washed again with low Ca^2+^ medium. The remaining vascular fragments were loaded with Ca^2+^-indicator by incubation in 1 ml of low Ca^2+^ Hanks’ solution containing 10 μM Fluo-4AM (Molecular Probes, Eugene, OR, USA) at 21 °C for 2 h. The suspension was then diluted with 10 volumes of low Ca^2+^ medium and vigorously triturated, and 1 ml of this mixture was pipetted into a circular glass-bottomed recording bath on the stage of an inverted microscope (Nikon Eclipse TE300). Microvessel segments between 0.2 and 2.0 mm in length, and with an outer diameter of 20–40 μm, were anchored down with tungsten wire slips and continuously superfused with normal Hanks’ solution at 37 °C. The recording bath was rotated to align the long axis of the arteriole to the *x*-axis of the microscope. Drug solutions were applied close to the tissue through a 5-way micro-manifold. The solution exchange time was approximately 1 s, as measured using a dye solution.

### 2.2 Solutions and drugs

The Hanks’ solution used to perfuse the bath had the following composition (mM): NaCl, 140; KCl, 5; d-glucose, 5; CaCl_2_, 2; MgCl_2_, 1.3; HEPES, 10; pH set to 7.4 with NaOH. The solution used for isolation of microvessels was of the same composition, but contained just 0.1 mM CaCl_2_. Stock solutions of drugs used were initially prepared as indicated below, and were diluted to the final concentration and applied in pre-warmed bath solution. The drug suppliers and bath concentrations of vehicle (%v/v) were as follows: cyclopiazonic acid (Alexis; 0.05% DMSO); ryanodine (Alomone; 0.1% ethanol), tetracaine (Sigma–Aldrich; 0.01% H_2_O), nifedipine (Sigma–Aldrich; 0.01% ethanol), EGTA (Lancaster; dissolved directly in bath solution).

### 2.3 Ca^2+^ imaging

#### 2.3.1 Imaging techniques

**Fig. 1:**
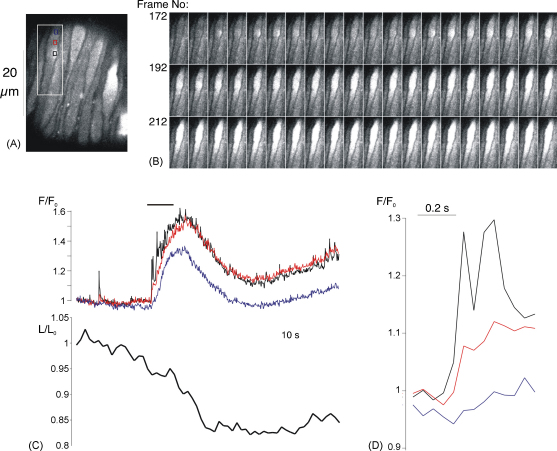
2D Ca^2+^-images from a retinal arteriole. (A) Single frame showing Fluo-4 fluorescence in an array of smooth muscle cells imaged in situ within a retinal arteriole. (B) Series of 60 consecutive frames (3 s of recording) for the area indicated by the white rectangle in A. Increased activity at the spark site is followed by a prolonged, global increase in [Ca^2+^]_i_. (C) The upper graph plots the normalised fluorescence against time for 3 regions of interest (ROIs) within the same cell (colour coded boxes in A). The period covered by the images in B is indicated by the horizontal bar above the graph. The lower graph plots changes in the length of this cell (normalisied to the initial length). The prolonged, global [Ca^2+^]_i_ increase was followed by a 10% decrease in cell length. (D) Changes in fluorescence for the three colour coded ROIs in A for frames 169–181.

Changes in intracellular [Ca^2+^] ([Ca^2+^]_i_) were imaged from adjacent smooth muscle cells in segments of intact arterioles. 2D Ca^2+^-images (Fig. 1) were obtained using a rotating-disc confocal system (QLC100, Visitech International) mounted on an inverted microscope (Nikon TE2000U) with a PlanApo, ×60, 1.4 NA, oil-immersion objective. A 488 nm LED-laser (Sapphire 488-20, Coherent) was used to excite the tissue. Emitted light was filtered (530–560 nm band-pass) and imaged using a high sensitivity, electron multiplying CCD camera (iXon, Andor Technology) at an acquisition rate of 20 frames per second. All other (1D linescan) experiments made use of a confocal scanning laser microscope (Bio-Rad, MR-A1) mounted on an inverted microscope (Nikon Eclipse TE300), again with a PlanApo, ×60, 1.4 NA, oil-immersion objective. Myocytes were scanned parallel to the long axis of the vessel (i.e., transversely across the cells) at a scan rate of 500 scans s^−1^. Emitted light was filtered (530–560 nm band-pass) and detected using a photomultiplier tube. Data acquisition was controlled with Timecourse software (Lasersharp™ add-on module; Biorad, US) and images were processed and analyzed with Image J (shareware, NIH, US). Fluorescence images were background corrected using an image frame collected without excitation. To allow for variations in dye loading, corrected confocal fluorescence data (*F*) were normalized using the average resting fluorescence (*F*_0_) for each cell, as measured during periods in which there was no increase in fluorescence. Changes in *F*/*F*_0_ were interpreted as changes in [Ca^2+^]_i_. Data collection was limited to a maximum of 80 s in linescan mode, after which background fluorescence started to increase due to photodamage. For presentation purposes, image brightness and contrast have been linearly adjusted for maximum clarity, with the same settings being used for all images from a given experiment.

#### 2.3.2 Event detection

Ca^2+^-events were detected using two criteria: the rate of rise of the signal and the amplitude of the event. Custom analysis software written in our laboratory by Dr. Norman Scholfield (V1.20, Borland Delphi, UK) used a gradient-change detection algorithm to identify rapid increases in fluorescence. The amplitudes of these events were recorded as the maximum increase in normalized fluorescence (Δ*F*/*F*_0_) above baseline. If the maximum increase in *F*/*F*_0_ was <2 × peak–peak resting noise, events were excluded from further analysis. Time dependent features for each event were measured using a plot of average fluorescence against time for a 5 pixel region of interest centered on the point of maximum fluorescence, and were summarized in terms of the time from event initiation to its peak (rise time), the time elapsed between half maximum amplitude fluorescence levels during the rising and falling phases, i.e., the full duration at half-maximal fluorescence (FDHM), the time between half-maximal fluorescence and the peak during the rising phase (half rise-time), and the time between the peak amplitude and half-maximal fluorescence during decay (half-time of decay).

### 2.4 Global [Ca^2+^] measurements

Global [Ca^2+^] measurements were recorded from the arteriolar smooth muscle using the Ca^2+^-indicator fura-2, as previously described [19,20]. Arterioles were incubated with 5 μM fura 2-AM for 2 h. They were then washed and superfused with Hanks’ solution at 37 °C in a perfusion bath mounted on the stage of an inverted microscope (Nikon Eclipse TE2000). Alternating excitation wavelengths of 340 and 380 nm light were delivered from a dual monochromator (5 nm bandwidth) using a light chopper (Cairn Research Ltd., Faversham, UK). Emitted fluorescence was measured from the side port of the microscope with an adjustable rectangular window, a filter (510 nm) and a photon counting photomultiplier tube (PMT) in the light path. Data acquisition, storage and analysis were controlled by Acquisition Engine (Cairn) software (V1.1.5). Changes in the ratio of fluorescenc emitted at each excitation wavelength (*R* = *F*340/*F*380) were used as a measure of changes in the cytoplasmic Ca^2+^ concentration.

### 2.5 Data analysis

**Fig. 2:**
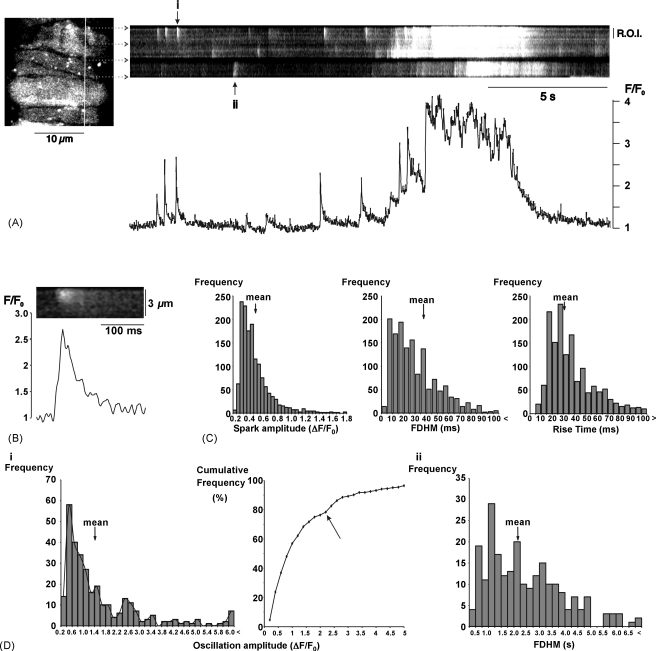
Spatiotemporal features of spontaneous Ca^2+^-sparks and oscillations. (A) The left hand panel shows smooth muscle cells imaged in situ within a segment of intact arteriole. The panel to the right shows a linescan image for 3 myocytes, as identified by the horizontal arrows. Changes in normalised fluorescence are plotted beneath the scan for the marked region of interest (R.O.I.) from the top cell. Note that the sparks marked ‘i’ and ‘ii’ spread across the most of the cell width. (B) The Ca^2+^-spark marked ‘i’ is shown on a faster time base. (C) Summary histograms for amplitude, full duration half maximum, and time to peak fluorescence for 1456 sparks recorded under control conditions in 158 cells within 38 arterioles. (D) Summary data for spontaneous oscillations recorded under control conditions from 183 cells within 27 arterioles. (i) Summary histogram and cumulative frequency plot for oscillation amplitude (304 events). The arrow marks a clear upward inflection in the cumulative frequency plot for amplitudes >2. (ii) Summary histogram for oscillation FDHM (233 events). Note that the time scale is in seconds, not ms as for the sparks.

Summarized data have been expressed as means ± S.E.M. Histograms for amplitude, rise time and FDHM for a large number of spontaneous Ca^2+^-events showed positive skew (Fig. 2C) and normality tests showed that the underlying frequency distributions were non-Gaussian (*P* < 0.0001 in each case, Kolmogorov–Smirnov test). The statistical significance of apparent differences between the mean values for amplitude, rise time and FDHM of these (unpaired) parameters before and during treatment in the same cell was assessed, therefore, using the Mann–Whitney *U*-test or non-parametric ANOVA (the Kruskal–Wallis test), with Dunn's multiple comparison test applied as a post hoc test when the ANOVA was significant. Given the high level of variability in events seen at different sites, comparisons of activity before and during treatment were all made for events occurring at the same site in the same cell before and during treatment, and only data from cells demonstrating spontaneous activity under control conditions were included. Paired data for the frequency of Ca^2+^-sparks and oscillations before and during treatment in the same cells were tested for statistical significance using the Wilcoxon signed-rank test or a non-parametric repeated measures ANOVA (Friedman test), again using Dunn's multiple comparison test where appropriate. The amplitudes of caffeine transients were compared using a parametric, repeated measures ANOVA, with Tukey–Kramer multiple comparisons post-hoc test. In all analyses the significance level was set at 0.05.

## 3 Results

### 3.1 Control observations of Ca^2+^-sparks and oscillations

In this study recordings were confined to arteriole segments that were 20–40 μm in diameter, which represent the main trunk arterioles that emanate from the optic disk. The wall of these vessels consisted of endothelium surrounded by a continuous layer of smooth muscle. This distinguishes these vessels from capillaries and venules, in which the outer layer of pericytes or muscle is discontinuous [21]. Spontaneous increases in intracellular [Ca^2+^] ([Ca^2+^]_i_) were routinely observed in myocytes within segments of intact retinal arterioles superfused with physiological solution at 37 °C (Figs. 1 and 2). These were subdivided into two main types: Ca^2+^-sparks, lasting less than 200 ms, and more prolonged events lasting several seconds (Figs. 1C, 2A and B). Control observations suggest that the more prolonged events may be initiated by Ca^2+^-spark activity. In 2D images, increased activity at Ca^2+^-spark sites often preceded more widespread, prolonged increases in [Ca^2+^]_i_, sometimes resulting in contraction (Fig. 1) [5]. Apparent summation of Ca^2+^-sparks to produce prolonged events was also seen on linescan images (Fig. 2). Both travelling Ca^2+^-waves and more synchronous Ca^2+^-oscillations were observed in 2D images (the prolonged event in Fig. 1 is an oscillation). As these events cannot be distinguished on transverse linescans, all prolonged events will subsequently be referred to as Ca^2+^-oscillations. The Ca^2+^-signal associated with sparks often spread across the cell width, and was not restricted to the near membrane space, as might be expected if there were a diffusion barrier (Fig. 2). This is consistent with the idea that sparks can contribute to a global elevation of [Ca^2+^]_i_ in arteriolar myocytes, causing contraction.

Descriptive statistics were summarized for a series of 1456 sparks recorded under control conditions from 158 cells in 38 vessels. The increase in normalised fluorescence (Δ*F*/*F*_0_) averaged 0.46 ± 0.01 (mean ± S.E.M.), with a mean time to peak fluorescence of 38.0 ± 0.5 ms, and a mean value for the full duration at half maximum (FDHM) of 30.6 ± 0.5 ms. Frequency distribution plots showed positive skew in all three parameters (Fig. 2C). Summary data has also been plotted for oscillations observed in 158 cells within 38 arteriole fragments. These had a mean amplitude of 1.42 ± 0.09 (304 events), and a mean FDHM of 2.20 ± 0.20 s (233 events). The frequency distribution for oscillation amplitude was bimodal, with a clear upward inflection in the cumulative frequency plot over the amplitude range 2.0–2.5 (Fig. 2D(i)). The distribution of FDHM also appeared multimodal, but it was not possible to identify the sub-populations of events. Overall, however, it would seem that Ca^2+^-oscillations do not represent a single population of events.

### 3.2 The SR Ca^2+^-ATPase and spontaneous Ca^2+^-events

**Fig. 3:**
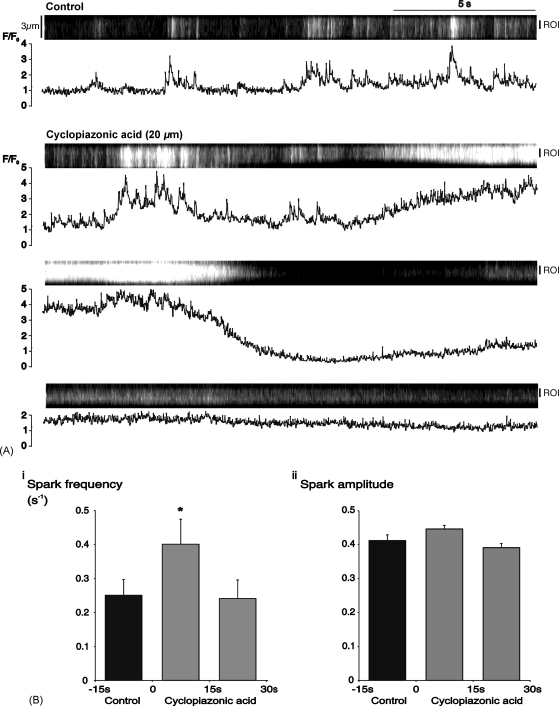
Cyclopiazonic acid initially increased Ca^2+^-spark frequency. (A) Linescan and graph showing control activity in a single myocyte within an arteriole under control conditions, and during the first 45 s of superfusion with 20 μM cyclopiazonic acid. Time-course data in the graph refers to the indicated region of interest (R.O.I.). (B) Summary data from 32 cells for, (i) spark frequency, and (ii) spark amplitude during 3 consecutive, 15 s periods (^*^P < 0.05 vs. control).

**Fig. 4:**
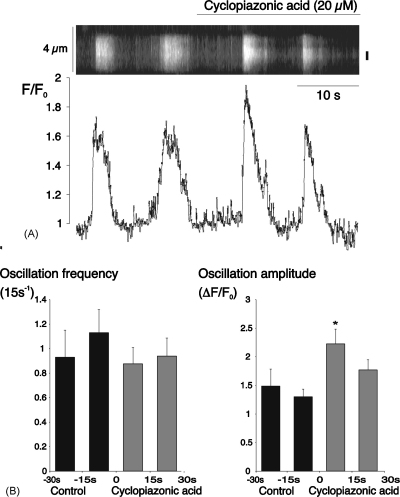
Cyclopiazonic acid initially increased the amplitude of spontaneous Ca^2+^-oscillations. (A) Linescan and normalized fluorescence plot for an arteriolar myocyte under control conditions and during superfusion with cyclopiazonic acid (20 μM). (B) Summary data (mean + S.E.M.) from 35 cells for 4 consecutive 15 s periods from 30 s before to 30 s after the start of superfusion with cyclopiazonic acid (^*^P < 0.05 vs. the second control period).

A series of experiments was carried out to determine the mechanisms responsible for the spontaneous Ca^2+^-signals seen in these vessels. Superfusion with cyclopiazonic acid (20 μM), an inhibitor of the Ca^2+^-ATPase in the SR membrane [22], initially increased spark activity (Fig. 3A). The mean spark frequency was increased from 0.25 ± 0.05 sparks s^−1^ during the last 15 s of the control period to 0.40 ± 0.07 s^−1^ during the first 15 s of cyclopiazonic acid treatment (*P* < 0.05; *n* = 32 cells in 3 vessels), but returned to control levels during the next 15 s (Fig. 3B(i)). There were no parallel changes in spark amplitude (Fig. 3B(ii)) or duration (data not shown). The increased spark frequency was associated with an increase in the amplitude of Ca^2+^-oscillations (Fig. 4A). Summary oscillation data from 35 cells in 3 vessels is plotted for 15 s time periods before and during treatment with cyclopiazonic acid (Fig. 4B). Mean amplitude increased from 1.30 ± 0.14 during the last control period to 2.22 ± 0.26 during the first 15 s period of cyclopiazonic acid treatment (*P* < 0.05), before decreasing back to control levels in the next 15 s.

**Fig. 5:**
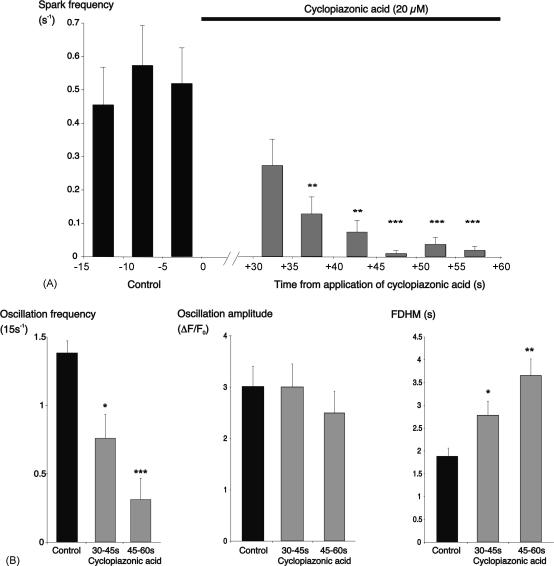
More prolonged application of cyclopiazonic acid inhibited both Ca^2+^-sparks and oscillations. (A) Summary bar chart for 22 cells showing spark frequency under control conditions and 30–60 s after start of exposure to cyclopiazonic acid. (B) Summary data for Ca^2+^-oscillations in the same experiments, showing changes in frequency, amplitude and full duration at half-maximum. Data was averaged over 15 s intervals (^*^P < 0.05; ^**^P < 0.01; ^***^P < 0.001; vs. the last control period in each case).

In a separate series of experiments, the effects of cyclopiazonic acid on sparks and oscillations were tested over a longer period of drug exposure. To avoid the effects of photobleaching, scanning was paused after a 20 s control period and recommenced 30 s after the beginning of superfusion with cyclopiazonic acid, allowing recording up to 60 s drug exposure. Spark frequency was decreased by this period of drug application, falling from 0.52 ± 0.11 sparks s^−1^ in the final 5 s control period, to 0.01 ± 0.001 s^−1^ after 50 s exposure to cyclopiazonic acid (*P* < 0.0001, *n* = 22 cells; Fig. 5). Spark amplitude and duration were unaltered. It should be noted that cyclopiazonic acid did not slow the rate of spark decay; indeed, the half-time of decay was slightly reduced from an average value of 14.2 ± 0.7 ms for sparks recorded under control conditions (*n* = 202 sparks recorded from 25 cells in 3 arterioles) to a mean of 12.2 ± 1.9 ms for sparks recorded in the same cells in the period from 30 to 60 s exposure to cyclopiazonic acid (*n* = 76 sparks; *P* < 0.0001). Oscillation frequency was also reduced in these experiments, falling from an average of 1.37 ± 0.09 (15 s)^−1^ during the last 15 s of the control period, to 0.30 ± 0.09 (15 s)^−1^ for the period from 45 to 60 s exposure to cyclopiazonic acid (*P* < 0.001, *n* = 30 cells; Fig. 5B). The mean duration of these oscillations increased from 1.88 ± 0.18 s during the control period to 3.65 ± 0.37 s during the last 15 s of drug application (*P* < 0.01, Fig. 5B). The mean oscillation amplitude, however, was not altered (Fig. 5B). Cyclopiazonic acid also elevated basal [Ca^2+^]_i_, with an average increase in overall fluorescence of 54 ± 12% after 45 s exposure to the drug (*P* < 0.0001, *n* = 31 cells).

These results indicate that release from the SR plays an important role in generating Ca^2+^-sparks and oscillations, so that when the store is depleted following prolonged blockade of the SR Ca^2+^-ATPase, this activity is inhibited. The next set of experiments was designed to identify the release mechanism responsible.

### 3.3 Ryanodine receptors and spontaneous Ca^2+^-events

Ca^2+^-sparks in other vascular smooth muscle preparations are generally agreed to result from the spontaneous opening of RyR gated channels in the SR [10,11]. Two pharmacological agents were used to test for this.

#### 3.3.1 Application of ryanodine

**Fig. 6:**
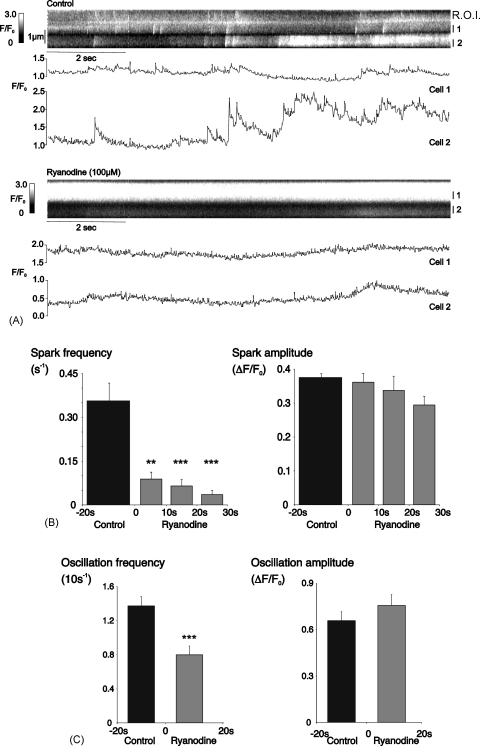
Ryanodine inhibited Ca^2+^-sparks and oscillations. (A) Linescan and normalised fluorescence plots for 2 adjacent myocytes in an arteriole under control conditions and during superfusion with 100 μM ryanodine. Time-course data in the graph refers to the indicated regions of interest (R.O.I.). (B) Summary data from 17 cells for spark frequency and amplitude during the 20 s control period and 3 consecutive, 10 s periods of superfusion with ryanodine (^**^P < 0.01; ^***^P < 0.001; vs. control). (C) Summary data from the same experiments for oscillation frequency and amplitude during a 20 s control period and the first 20 s superfusion with ryanodine (^**^P < 0.01; ^***^P < 0.001; vs. control).

A high concentration of ryanodine (100 μM) was used in an attempt to minimise the number of RyRs locked open in a sub-conductance state, as this could produce non-specific reduction in Ca^2+^-release by any pathway secondary to store-depletion [23]. This concentration has been used to block RyRs in other recent studies [24,25]. Superfusion with ryanodine inhibited both Ca^2+^-sparks and oscillations, as shown in the linescan and time-plot from 2 adjacent cells in a single arteriole (Fig. 6A). The average spark frequency fell from 0.36 ± 0.06 s^−1^ during the last 20 s of the control period, to 0.04 ± 0.02 s^−1^ after 20 s superfusion with ryanodine (*P* < 0.0001, *n* = 17 cells, 4 arterioles). Oscillation frequency was reduced from 1.37 ± 0.11 (10 s)^−1^ under control conditions to 0.80 ± 0.11 (10 s)^−1^ during the first 20 s of ryanodine application (*P* < 0.001). In those cells in which spontaneous activity persisted in the presence of ryanodine, there were no statistically significant changes in mean amplitude or duration for either sparks or oscillations.

#### 3.3.2 Application of tetracaine

**Fig. 7:**
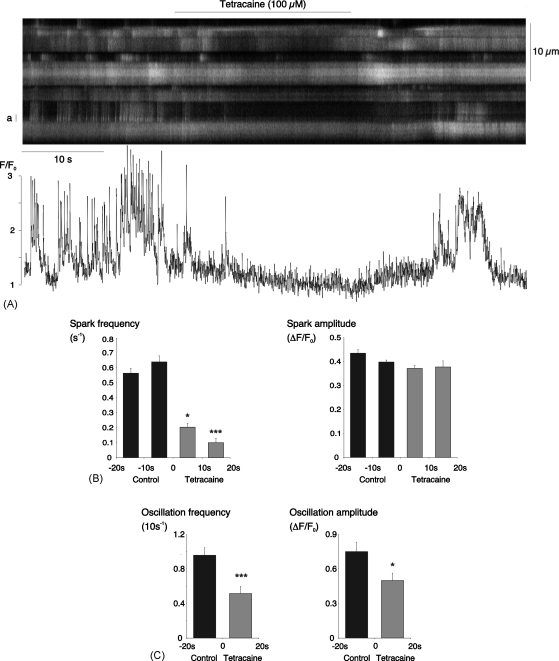
Tetracaine inhibited Ca^2+^-sparks and oscillations. (A) Linescan of 9 adjacent myocytes in an arteriole under control conditions, during superfusion with 100 μM tetracaine and during the subsequent washout. A time plot of the normalised fluorescence for the cell marked ‘a’ is shown beneath the scan. (B) Summary data from 29 cells for spark frequency and amplitude during two 10 s control periods and two, 10 s periods of superfusion with tetracaine (^*^P < 0.05; ^***^P < 0.001; vs. the 2nd control period). (C) Summary data from the same experiments for oscillation frequency and amplitude during a 20 s control period and the first 20 s superfusion with tetracaine (^*^P < 0.05; ^***^P < 0.001; vs. the control period).

To further test for RyR-involvement, we applied tetracaine, an inhibitor of RyRs with no known store-depleting action [26,27]. Superfusion with 100 μM tetracaine rapidly and reversibly inhibited both sparks and oscillations (Fig. 7A). In data from 29 cells in 12 arterioles, the mean spark frequency was reduced from a control value of 0.66 ± 0.11 to 0.10 ± 0.03 s^−1^ for the period from 10 to 20 s after the start of tetracaine superfusion (*P* < 0.001, Fig. 7B). In the same experiments, the average oscillation frequency was decreased from 1.91 ± 0.18 (10 s^−1^) during control to 1.06 ± 0.16 (10 s^−1^) in the presence of tetracaine (*P* < 0.001, Fig. 7C). There was no change in spark amplitude but there was a decrease in the mean amplitude of Ca^2+^-oscillations, which fell from 0.75 ± 0.08 for 65 oscillations recorded under control conditions to 0.50 ± 0.06 for 36 oscillations recorded in the presence of tetracaine (*P* < 0.05).

Taken together the effects of ryanodine and tetracaine strongly suggest that the spontaneous Ca^2+^-events seen in the arteriolar smooth muscle result from opening of RyR-gated Ca^2+^-release channels.

### 3.4 Ca^2+^-influx and spontaneous Ca^2+^-events

Influx of Ca^2+^ across the plasma membrane could play a role in the generation of spontaneous Ca^2+^-release events in at least two distinct ways; by triggering the opening of the Ca^2+^-sensitive Ca^2+^-release channels in the SR [23,28], or by replenishing the Ca^2+^-store itself [29]. This was investigated both pharmacologically and by removal of external Ca^2+^.

#### 3.4.1 Application of nifedipine

**Fig. 8:**
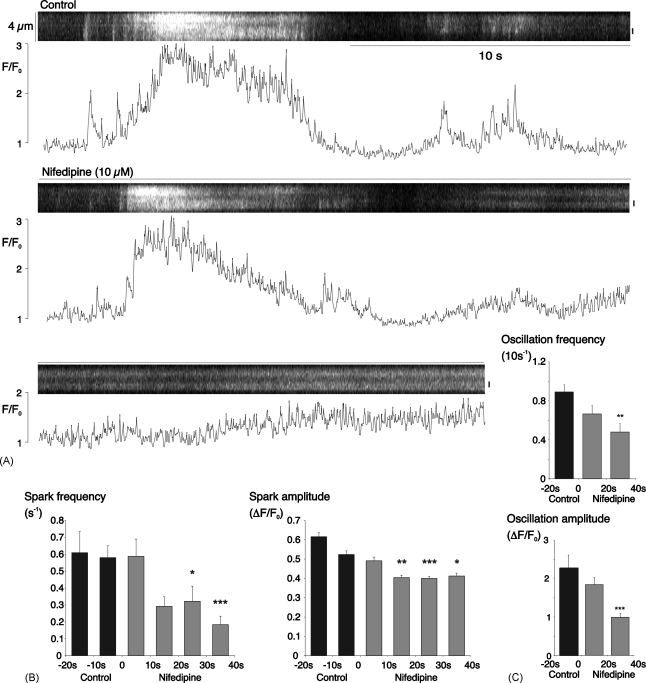
Nifedipine partially inhibited Ca^2+^-sparks and oscillations. (A) Linescan and normalised fluorescence (for the region marked by a vertical bar to the right) under control conditions and during superfusion with 10 μM nifedipine. (B) Summary data from 24 cells for spark frequency and amplitude during two 10 s control periods and the first 4, 10 s periods of superfusion with nifedipine (^*^P < 0.05; ^***^P < 0.001; vs. the second control period). (C) Summary data from the same experiments for oscillation frequency and amplitude during a 20 s control period and the first two, 20 s periods of superfusion with nifedipine (^**^P < 0.01; ^***^P < 0.001; vs. the control period).

Nifedipine, long recognized as an inhibitor of high-voltage activated, L-type Ca^2+^-channels in smooth muscle (reviewed in [30]), has recently been shown to inhibit Ca^2+^ store-refilling by another mechanism in both choroidal and retinal arterioles [29,31]. Nifedipine (10 μM) reduced both sparks and oscillations (Fig. 8). Summary data for 24 cells in 4 arterioles shows that the mean spark frequency was reduced from an average control value of 0.58 ± 0.07 to 0.13 ± 0.04 s^−1^ after 30 s exposure to nifedipine (*P* < 0.001, Fig. 8B). Mean spark amplitude was also decreased from 0.52 ± 0.02 for 139 control sparks, to 0.40 ± 0.01 for 71 sparks recorded during the period from 20 to 30 s superfusion with nifedipine (*P* < 0.001). Oscillation frequency was reduced from a mean control value of 0.89 ± 0.07 (10 s^−1^) to 0.48 ± 0.09 (10 s^−1^) between 20 and 40 s nifedipine exposure (*P* < 0.01). Average amplitude fell from 2.27 ± 0.34 for 37 oscillations recorded under control conditions to 0.99 ± 0.11 in 37 oscillations recorded from 20 to 40 s after the start of nifedipine application (*P* < 0.001).

#### 3.4.2 Removal of external Ca^2+^

**Fig. 9:**
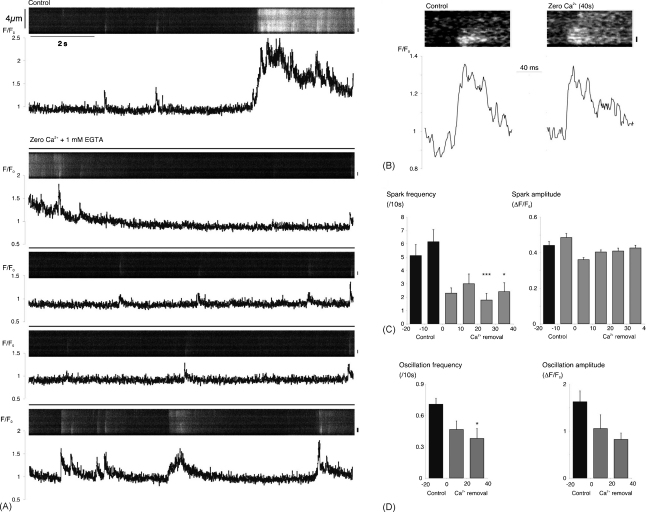
Low external [Ca^2+^] rapidly reduced but did not abolish spontaneous activity. (A) Linescan and normalised fluorescence plot for a myocyte under control conditions and during superfusion with a low [Ca^2+^] test solution. The region of interest is indicated by the vertical bar to the right of each image. (B) Fast time-base linescans of two sparks recorded at the same site in this cell, both during the control period and after 40 s of superfusion with the test solution. (C) Summary data from 28 cells for spark frequency and amplitude during two 10 s control periods and the first 4, 10 s periods of superfusion with low [Ca^2+^] solution (^*^P < 0.05; ^***^P < 0.001; vs. the 2nd control period). (D) Summary data for oscillation frequency and amplitude in the same cells during a 20 s control period and the first two, 20 s periods of superfusion with the test solution (^*^P < 0.05 vs. control).

Although nifedipine reduced spontaneous activity, it did not abolish it over a period of 40 s. Incomplete inhibition of activity may have reflected incomplete blockade of Ca^2+^-influx across the plasma membrane. In order to test this more directly, a low [Ca^2+^] bathing solution was used, in which CaCl_2_ was substituted with MgCl_2_, and 1 mM EGTA was added as a Ca^2+^-chelator. Although this produced a reduction in spark frequency and amplitude over the first 20 s of superfusion in the sample record shown, activity recovered somewhat with continued superfusion so that, after 40 s superfusion, spark amplitude and timecourse were similar to control, even though spark frequency remained depressed (Fig. 9). In a series of 8 such experiments, spark frequency was reduced from an average control value of 0.62 ± 0.09 s^−1^ in 28 cells, to 0.18 ± 0.05 s^−1^ from 20 to 30 s after the start of perfusion with the low [Ca^2+^] solution (*P* < 0.001, Fig. 9C). There were no statistically significant changes in mean spark amplitude (Fig. 9C) or duration (data not shown). Oscillation frequency was also reduced in these experiments from an average control value of 0.71 ± 0.06 (10 s^−1^) to a mean of 0.38 ± 0.10 (10 s^−1^) during the second 20 s period of superfusion with low [Ca^2+^] (*P* < 0.05, Fig. 9D). The persistent but reduced level of activity in the presence of nifedipine or low external [Ca^2+^] suggests that influx across the plasma membrane largely plays an indirect role in the generation of spontaneous Ca^2+^-events, although direct coupling of influx to spark activity through calcium-induced calcium-release cannot be excluded.

### 3.5 Changes in Ca^2+^-store content

**Fig. 10:**
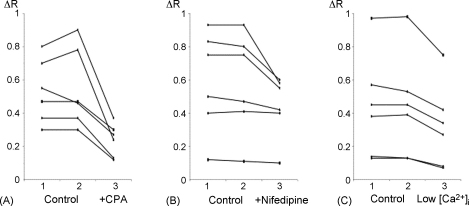
Cyclopiazonic acid, nifedipine and low external [Ca^2+^] reduced but did not abolish caffeine transients. (A) Increases in fura2 fluorescence ratio (Δ*R*) evoked by 3 serial applications of caffeine (10 mM) in 6 different arterioles. The first 2 applications (1 and 2) were made under control conditions and produced similar responses. The third application (3) was made after 40 s superfusion with cyclopiazonic acid (20 μM), and this response was reduced in each case. (B) Similar plots for 6 arterioles comparing 2 control applications of caffeine with a third application after 40 s exposure to nifedipine (10 μM). (C) Similar plots for 6 arterioles comparing 2 control applications of caffeine with a third application after 40 s exposure to low external [Ca^2+^].

We have suggested that the reductions in spontaneous activity seen following inhibition of the SR Ca^2+^-ATPase or Ca^2+^-influx across the plasma membrane may result from reductions in SR Ca^2+^-loading. Store content was investigated more directly by recording caffeine-evoked Ca^2+^-transients from arterioles loaded with fura-2. Caffeine (10 mM) was applied twice under control conditions in each experiment, and was then applied to the same preparation for a third time after 40 s of the relevant treatment (Fig. 10). Control responses were highly repeatable, but caffeine transients were reduced in amplitude following 40 s superfusion with cyclopiazonic acid (20 μM; *n* = 6, *P* < 0.001; Fig. 10A), nifedipine (10 μM; *n* = 6, *P* < 0.05; Fig. 10B), or low [Ca^2+^] solution (1 mM EGTA; *n* = 6, *P* < 0.001; Fig. 10C). It is clear, however, that the stores still contained considerable releasable Ca^2+^ at these times, as caffeine still evoked substantial transients. This suggests that reductions in RyR open probability resulting from reduced SR [Ca^2+^] may play a more important role in reducing spontaneous activity than the decline in the driving force for Ca^2+^-release.

## 4 Discussion

### 4.1 Functional significance of sparks and oscillations

#### 4.1.1 Spark summation can generate prolonged oscillations

This report confirms our preliminary observations in this preparation and extends them to consider the mechanisms responsible for the spontaneous Ca^2+^-events seen [7]. Brief sparks and more prolonged oscillations were observed in 2D images (Fig. 1) and in transverse linescans (Fig. 2). Global oscillations, which were associated with mycoyte contraction, often appeared to result from the summation of Ca^2+^-sparks in linescan images (e.g., Figs. 1, 2A, 3A, 7A). These observations suggest a possible role for spark summation as a mechanism for vasoconstriction. It was also noted that Ca^2+^-sparks, which often originated near the plasma membrane, could increase [Ca^2+^]_i_ across the width of the cell (e.g., Fig. 2). They did not appear to be restricted to the near-membrane space, although the concentrations achieved in this region must be much higher than elsewhere. The existence of a diffusion- or buffer-barrier has been proposed in a variety of smooth muscles [32–35]. The absence of any obvious diffusional barrier, along with the relatively small size of the myocytes themselves, may facilitate elevation of global [Ca^2+^]_i_ to the levels necessary for contraction through spark summation.

If Ca^2+^-sparks promote Ca^2+^-oscillations, then interventions which modulate spark activity should produce parallel changes in the amplitude or frequency of oscillations. This expectation was fulfilled both under conditions in which spark activity was increased (Figs. 3 and 4), and during five different inhibitory protocols (Figs. 5–9). The simplest interpretation of the experimental results is, therefore, that similar mechanisms are responsible for both phenomena. This suggestion, in which sparks can have an excitatory role through generation of waves or oscillations, contrasts with the inhibitory role often ascribed to them in vascular and other smooth muscles, as first demonstrated in cerebral arteries [14]. The high near-membrane [Ca^2+^] achieved during such localised events can activate Ca^2+^-sensitive K^+^-channels [36,37], polarising the cell and reducing Ca^2+^-influx by de-activating voltage sensitive Ca^2+^-channels [15–18]. It appears, however, that this may not be the only function of sparks within retinal arterioles, even though these myocytes do express Ca^2+^-activated K^+^-current [38]. The proposal that sparks can act as building blocks for Ca^2+^-waves and oscillations in retinal arterioles is consistent with observations from smooth muscle cells isolated from mesenteric arteries and portal veins, in which global signals were often observed to originate from foci of high frequency spark activity, dubbed ‘frequent-discharge-sites’ [39,40]. The details of feedback control via voltage-activated Ca^2+^-channels in arteriolar preparations remain to be determined.

#### 4.1.2 Frequency modulation of sparks

The current data supports a model in which sparks within retinal arterioles behave as stochastic, frequency-modulated signalling events, from which more global and prolonged signals can be constructed. The mean amplitude of the sparks was little affected under a variety of conditions in which spark frequency was greatly altered (Figs. 3, 5–7 and 9). Depolarisation can increase spark frequency with little, if any, effect on spark amplitude in both smooth and striated muscle [37,41]. Previous studies on vascular myocytes have also reported an increase in spark frequency with no parallel increase in amplitude during chronic and acute store overload [42,43]. In the latter report, spark amplitude was reduced following store depletion with thapsigargin, but mean spark amplitude only fell by 25% under conditions in which the caffeine-evoked transient was diminished by almost 60%. In the current experiments, 60 s superfusion with cyclopiazonic acid almost abolished spark activity but had no effect on the amplitude of the small number of residual sparks seen (Fig. 5). The reduced frequency may reflect a fall in the open probability of the release channels when [Ca^2+^] on the luminal side of the channel is decreased [44]. Although caffeine-evoked [Ca^2+^] transients were consistently reduced in size after 40 s exposure to cyclopiazonic acid, nifedipine or low [Ca^2+^] solution (Fig. 10), there was a substantial residual transient in each case. This amounted to 45 ± 6% of control in the case of cyclopiazonic acid, 81 ± 6% of control for nifedipine, and 69 ± 4% of control with low [Ca^2+^]. This implies that there was still a substantial gradient driving Ca^2+^-release in each case, consistent with the notion that the decline in spontaneous activity seen under each of these conditions reflected failure of channel opening rather than inadequate flux through open channels.

### 4.2 The role of the SR in sparks and oscillations

#### 4.2.1 SR filling via the SRCa-ATPase sustains Ca^2+^-sparks and oscillation

Inhibition of Ca^2+^-uptake into the SR, resulting in store depletion, ultimately reduced spontaneous activity (Fig. 5). The initial response to cyclopiazonic acid, however, involved an increase in spark frequency (Fig. 3), possibly reflecting a local increase in cytoplasmic [Ca^2+^] in response to reduced store uptake. Any such increase could promote activation of Ca^2+^-sensitive release channels [45], resulting in the observed increase in the amplitude of oscillations (Fig. 4). It is possible, however, that the short-lived increase in oscillation amplitude may have resulted directly from decreased re-uptake into the SR, leading to a greater increase in cytoplasmic [Ca^2+^] for a given influx or release. The subsequent reduction in the size and frequency of oscillations, indicates, however, that slowed removal alone cannot explain all the changes seen, and suggests that store depletion is ultimately responsible for the decay in activity, as previously described in arterial muscle treated with thapsigargin [43].

It is instructive to compare the effects of cyclopiazonic acid on sparks and oscillations, since the changes in spark frequency were not associated with any changes in spark amplitude or kinetics during the initial period of increased activity (Fig. 4), or its subsequent inhibition (Fig. 5). This suggests that, unlike sparks in cardiac muscle [46], the decay phase is not appreciably dependent on re-uptake by the SR in retinal arteriolar myocytes. Again, this is consistent with previous studies in which thapsigargin had no effect on the decay of sparks in arterial muscle, despite reducing spark frequency and, after 5–15 min exposure, spark amplitude [43]. It may seem counterintuitive that cyclopiazonic acid slowed the decay of oscillations but not that of sparks. The time constant of [Ca^2+^] decay due to uptake by the SR Ca^2+^-ATPase in cardiac microsomes is, however, of the order of 20 s at 37 °C [47]. Even allowing for optimisation of pump activity within the cell, removal mechanisms with relatively slow kinetics like this are much more likely to play a role in shaping prolonged events than sparks lasting approximately 100 ms.

#### 4.2.2 Spontaneous Ca^2+^-events result from ryanodine receptor activation

As in other smooth and striated muscles, activation of ryanodine receptors plays a crucial role in spark generation within these arterioles (reviewed in [1–3]). Sparks were inhibited both by ryanodine and tetracaine (Figs. 6 and 7). A high ryanodine concentration was used deliberately [24,25] and it seems unlikely that its action can be dismissed as a non-specific consequence of store-depletion [48]. Prolonged activation of a subconductance state of the ryanodine receptor, which might cause such depletion, would be expected to elevate mean ‘resting’ [Ca^2+^]_i_, and yet inhibition was seen both in cells which showed an increase and in those demonstrating a decrease in basal cytoplasmic [Ca^2+^] (Fig. 6A). It seems unlikely that these changes in basal fluorescence resulted from movement artefact since no changes in cell dimensions were seen, although some rotation about the long axis of the myocytes cannot be excluded. The response to 100 μM tetracaine, which reduces RyR open probability leading to an increase in SR [Ca^2+^] in cardiac and smooth muscle [43,49], was rapid, and inhibition of spontaneous activity was complete and reversible in many cells (Fig. 7). It is unclear why oscillation amplitude was reduced by tetracaine but not by ryanodine (Figs. 6C and 7C).

Taken together, these findings indicate that RyR-channel activation is a key event in the spontaneous Ca^2+^-signals seen in these cells. What remains unclear, however, is how the open probability of the release channels is modulated over time, as reflected in the observation of both isolated release events and the summation of events to give more prolonged oscillations at different times at the same site in a given cell (Fig. 2A). It can be seen from some of the examples shown that this was not obviously correlated with the spark amplitude, as large, single sparks were often followed by oscillations initiated by a smaller spark at the same site (e.g., Figs. 2A, 6A and 9A). Modulation of the open probability of ryanodine channels by factors such as channel phosphorylation, variations in [Ca^2+^] within the SR, or the cytoplasmic concentration of other second messengers, e.g., inositol 1,4,5-trisophosphate or cyclic ADP-ribose (see reviews: [3,45,50]), may well play a role but the results presented here cast no light on this important issue.

### 4.3 Reduced Ca^2+^-influx partially inhibited sparks and oscillations

Inhibition of influx of extracellular Ca^2+^ reduced spontaneous Ca^2+^-signalling both in the presence of nifedipine and when extracellular Ca^2+^ was removed (Figs. 8 and 9). Nifedipine's inhibitory action in the current experiments need not be interpreted as indicating a substantial role for influx mediated by L-type Ca^2+^-window current under resting conditions, as store operated channels in rat choroidal and retinal arterioles are also nifedipine sensitive [29,31]. It was notable that spark generation was maintained at a reduced but relatively constant frequency up to 40 s in both conditions, whereas tetracaine, which directly inhibits ryanodine channel opening, produced 80% blockade within 20 s (Fig. 7). This suggests that spark production is not tightly coupled to influx, as it is believed to be in cardiac muscle [51], and is consistent with previous reports that ryanodine receptor activation is loosely linked to voltage activated Ca^2+^-influx in smooth muscle [52]. It would appear, however, that Ca^2+^-entry pathways do play an important role in determining basal activity in these vessels, possibly by refilling the SR stores (Fig. 10). Identifying and characterising the relevant pathways both molecularly and pharmacologically remains an important goal for future work. Surprisingly, reducing external [Ca^2+^] had no effect on spark amplitude but nifedipine decreased it, even though both treatments produced similar reductions in store content (Fig. 10). The drop in spark amplitude was relatively small (approximately 20%), however, and its biological importance is unclear.
